# Autopsies at Central Park Menagerie

**Published:** 1885-07

**Authors:** 


### CASE DEPARTMENT.

### I.—AUTOPSIES AT CENTRAL PARK MENAGERIE.

*Axis Deer* (*cervus axis Erxl*), Weight 75 pounds.—Made two days after death. Body already skinned, decapitated and without limbs. Owing to the objections raised by the osteologist of the institution against the skull being properly opened the brain could not be examined.

*Heart.*—Weighed eight ounces; thickness of wall of left ventricle one inch, that of right ventricle, three-eighths of an inch; all the cavities were filled with dark red and whitish clots, which also extended into the large vessels immediately adjoining the organ; its color was of a dark brownish red.

*Lungs and Pleura*.—Parietal layers of pleura adhering firmly to chest walls; upper lobe of left lung decidedly emphysimatous and cavernous, of a light brown anaemic appearance; lower lobe of a dark brownish-red color, easily broken and friable; blood vessels overdistended with blackish blood, a mixture of frothy mucous and blood oozing out from the cut surfaces; tuberculous; miliary tubercle also found over pleural surfaces. Right lung in same condition. Weight, left, eight ounces; right, twelve and a-half ounces.

*Spleen.*—Length, six and a-half inches; breadth, three inches; thickness, three-quarter inch; surface, steel gray; pulp, brownish-red in color, weighed two ounces six drachms.

*Kidneys.*—Capsules easily removed, surface of a steel gray color; parenchyma of a brownish-red color; otherwise normal in appearance; right weighed one ounce seven drachms; left, two ounces.

*Liver.*—Strongly adherent to diaphragm; weighed one and a-half pounds; longest diameter, ten and a-half inches; short diameter, six ounces; thickness, two and a-half inches; of a normal dark brownish red color.

*Intestines.*—Both rumen and reticulam partially filled with boluses of undigested vegetables, showing marked signs of engorgement; vessels, prominent and ingested; all the rest of the intestinal canal was, throughout, covered with flakes of recent lymph and the great omentum and the mesenteries adhered together and to the abdominal parietes; it was due to this that the length of the canal could not be accurately determined. Miliary tubercles found on omentum and mesenteries. On about the centre of the great omentum a small cyst was discovered, of the size of a pigeon's egg, and of a yellowish, perfectly transparent color, with semi-fluid, gelatinous contents adhering to the omentum by a short pedicle. All the viscera showed well-marked signs of congestion and recent inflammation.

*Bladder.*—Contracted in pelvis and normal.

*Cause of death.*—Tuberculosis; immediate cause, tuberculor peritonitis.

H. G. BEYER, M.D., U. S. Navy.

*Dorcas Gazelle* (*Kobus gazella dorcas*).—Had convulsions April 18th, 1885, in one of which it died; supposed to have been brought on by indigestion. Head, extremities and skin of trunk had been removed. Decomposition somewhat advanced: larynx contained two large blood clots, each about two inches long and several lines in diameter, one extremity of one of which protruded from glottis; source of hæmorrhage not apparent. Lungs showed small extravasations. Heart contained soft dark clots. Rumen filled with three pounds of oats and hay undigested. Gall-bladder empty; spleen seemed shrunken; small intestines empty; large intestines contained some flatus; cortical substance of kidneys very dark. Cause of death probably asphyxia from closure of the glottis by blood-clot.

F. W. TRUE, Curator, U. S. Museum.

*Water Buck* (*Kobus ellipsiprymnus.*)—Decomposition well advanced; mucous membrane of larynx and trachea nearly black; pleuro-pericardium and peritoneum contained much bloody serum; lungs, collapsed; heart, flabby, full of black blood and clots; œsophagus and rumen distended with undigested food; intestines, empty; greater omentum, lower free margin, showed a cyst containing a vesicular entozoon, two inches long, with head flattened laterally and single central mouth; not yet identified. Some bile in the gall-bladder. Cause of death not clear, probably over-distension of stomach.

F. W. TRUE, Curator, U. S. Museum.

